# Has the development of cancer biomarkers to guide treatment improved health outcomes?

**DOI:** 10.1007/s10198-021-01290-4

**Published:** 2021-03-30

**Authors:** Ana Beatriz D′Avó Luís, Mikyung Kelly Seo

**Affiliations:** 1 Department of Economics, University of Bergen, Fosswinckelsgate 14, 5007 Bergen, Norway; 2 Centre for Cancer Biomarkers (CCBIO), Faculty of Medicine, University of Bergen, Bergen, Norway; 3 Department of Health Services Research and Policy, Faculty of Public Health and Policy, London School of Hygiene and Tropical Medicine, London, UK; 4 Department of Surgery and Cancer, Faculty of Medicine, Imperial College London, London, UK

**Keywords:** Biomarker, Personalized medicine, Cancer, Mortality, I11, O33

## Abstract

During the last decade, testing the patient’s biomarker status prior to the administration of corresponding co-dependent therapies has been emerging in clinical practice. These biomarker-guided therapies have promoted the promise of more personalized medicine, with the prescription of the right treatment to the right patient, while avoiding expensive ineffective drugs and adverse drug reactions. Cancer treatments have especially taken advantage of this technology. We assess how the introduction of biomarker tests guiding cancer therapy have affected the premature mortality and survival of cancer patients in Norway. Our findings suggest that, in general, cancer patients have benefited from both biomarker testing and more cancer drugs. Furthermore, we find that the total effect of biomarker testing on 3-year survival decreases as the number of drugs available increases, suggesting that the matching of patients with the appropriate treatment is better when fewer drugs are available.

## Introduction

Since the sequencing of the human genome in 2001, personalized medicine through the use of biomarker-guided therapies—that is, those drugs for which response can be predicted by a biomarker test—has been the subject of discussion within the pharmaceutical industry and health policy research. Although this technology is promising, it has made slower-than-expected progress. Several factors might contribute to this phenomenon, including the lack of appropriate economic incentives for pharmaceutical firms, the differences in regulatory approval between countries, and the complexity of the science and imprecision of biomarker prediction [10]. This study focuses on the importance of the latter and asks the following question: has the incorporation of biomarker tests to predict drug response played a role in the improvement of the health of patients diagnosed with complex diseases such as cancer?

Regulatory authorities such as the U.S. Food and Drug Administration (FDA) and the European Medicines Agency (EMA) have been actively encouraging the use of biomarker tests in the development and use of prescription drugs [23]. The Directorate of Health in Norway has developed a national strategy for the implementation of personalized medicine in health care services. Its aim is to have more personalized medicine available, ensuring the right treatment for the right patient at the right time [22]. In this context, more personalized medicine can be achieved through the use of biomarker tests that look for molecular mutations or gene amplification to identify the group of patients more likely to respond to a specific treatment.

Biomarker testing has allowed for new approaches to diagnosis and treatment. It can assist decision-making by optimizing treatment for the right patients who will be responsive to the corresponding therapy, while providing guidance on excluding patients deemed unlikely to respond. Therefore, in addition to the direct effects of treatment, it has the potential to reduce adverse drug reactions and overtreatment, and hence may improve the health and quality of life of patients. Additionally, it can potentially limit the expenditure on ineffective therapies and create more sustainable health care systems.

Despite the increased adoption of this technology, especially in oncology, progress has been slower than expected, and very few biomarkers reach clinical practice. One potential reason is the limitations of genetic prediction, mainly due to biological complexity, which affects the predictive capacity of the biomarker and limits the evidence of health impact. This scientific challenge to the development of effective drug–test combinations has been pointed out in both the medical and the health economics literature [9, 24]. Moreover, it is still unclear whether the use of biomarker tests for cancer therapies has improved health outcomes for cancer patients.

Analyzing the health impact of biomarker tests on long-term health outcomes is crucial to obtain appropriate coverage and to prioritize between innovations [24]. Testing biomarker status prior to the administration of the corresponding treatment is potentially a way to avoid waste of public funds on harmful or ineffective drugs. Thus, the focus for the health authorities should be on facilitating regulation for the development and approval of cost-effective personalized medicine. Therefore, it is of importance to assess the impact of the clinical use of biomarkers on patient outcomes as a whole and inform stakeholders regarding their value for money. Although there are some studies on the effect of personalized medicine, they may not entirely reflect the reality of clinical practice. Most existing evaluations of cancer biomarkers are individually conducted using respective clinical trial data or simulations based on evidence from clinical trials (for instance, [2] for KRAS and BRAF in colorectal cancer, and [26] for EGFR in lung cancer). However, clinical trials can be executed in highly controlled settings or on small samples. Pharmaceutical companies that may have an interest in showing the benefits of their new medicine may carefully select the participant patients, for example with relatively uncomplicated cases [31]. It is, therefore, a paradox that there are fewer restrictions in actual routine practice where biomarkers are likely to be used differently, compared to settings that closely follow clinical guidelines, such as clinical trials or academic environments [24]. Moreover, few evaluations are performed at the system or population level using real-world longitudinal and/or patient-level data.

Previous work has documented the effect of pharmaceutical innovation for cancer on increasing survival or reducing mortality. For example, [14, 15, 17–21] has made difference-in-differences estimates using panel data for different cancers for a range of countries, including the United States, France, Canada, Switzerland, Belgium, and Mexico. His results suggest that cancer types that experienced an increase in the number of pharmaceutical products had larger increases in life expectancy [14, 15] and survival rate [21], larger declines in premature mortality [17–19, 21], and larger reductions in mortality rates [15, 20]. Dubois and Kyle [5] use panel data from 11 countries to study this issue and find that cancer mortality declines with the availability of additional treatments. These studies are based on a theoretical model of endogenous technological change developed by Romer [27]. The idea is that an economy’s output depends on the stock of ideas that have previously been developed. The models estimated can be considered a health production function, in which the health outcome (survival, mortality rate, or premature mortality) depends on the cumulative number of treatments approved.

In this paper, we seek to provide new insights concerning the relationship between the use of cancer therapies with biomarker tests and health outcomes using Norwegian data. The aim is to determine the effect of the utilization of biomarkers for cancer therapies on premature mortality and survival. The analysis is performed based on the availability of cancer treatments and on individual-level data covering all cancer diagnosis and cancer-related deaths in Norway from 2000 to 2016. During this period, a few biomarker-guided therapies were introduced for the treatment of some types of cancer. To analyze how biomarker tests affected premature mortality and survival probabilities, we make use of the fact that they were introduced for different cancers at different points in time. We also use the variation in the number of biomarker-guided drugs launched to investigate whether cancers that had more guided drugs available had larger declines in potential years of life lost or larger increases in the probability of surviving 3 years after diagnosis.

This paper adds to the literature by analyzing the effect of biomarker tests on cancer treatment, using detailed registry data on cancer patients. The baseline empirical strategy is to regress health outcomes (potential years of life lost before age 75 and 65, and a 3-year survival dummy variable) on the number of cancer drugs and the availability of biomarker tests to treat the specific cancer each patient is diagnosed with. Potential years of life lost is an indicator of cancer premature mortality and can capture the effect of biomarkers and drugs available over the patient’s full period of treatment, whereas probability of survival three years after diagnosis mainly captures the effect of the biomarkers and drugs available at the beginning of the treatment period. An advantage of premature mortality over survival probability is that the former is not subject to lead-time bias.^1^

After controlling for general time-varying aspects, our findings suggest that having at least one biomarker test available decreases premature mortality on average, and the total effect of biomarker testing on survival decreases as the number of cancer drugs available increases. This result suggests that biomarker tests improve health by better matching patients to treatment, but that matching is better when fewer drugs are available. Furthermore, we find that nonguided therapies, which do not require biomarker testing, are associated with an increased probability of being alive 3 years after diagnosis, while biomarker-guided drugs, which require biomarker testing, are associated with a reduction of premature mortality before age 75 and 65. Estimates of the cost per life-year gained before ages 75 and 65 in 2016 from biomarker-guided drugs introduced during 2000–2015 are NOK 64,212 and NOK 72,517, respectively. These figures are below the threshold value used in the literature at which an intervention is considered cost-effective [25].

This article will proceed as follows. Section 2 presents the setting of cancer treatment in Norway; in Sects. 3 and 4, the data sources and methodology are described. In Sect. 5, the results of the empirical analysis are reported, and in Sect. 6, a robustness check is shown to validate the main results of the analysis. Section 7 discusses the main results and presents the main shortcomings; Sect. 8 concludes.

## The setting of cancer treatment and biomarker use in Norway

**Fig. 1:**
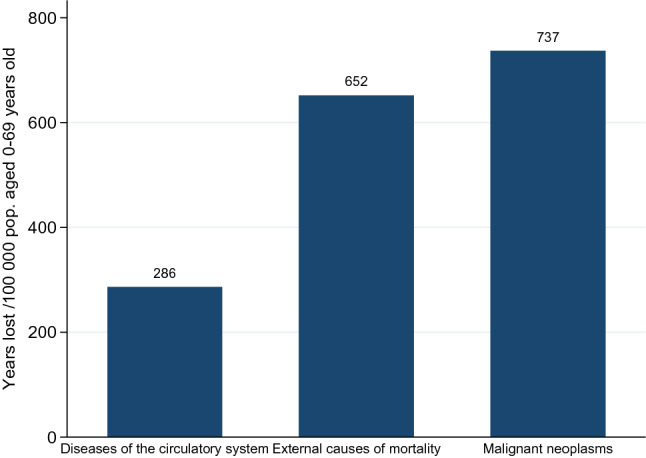
Premature (before age 70) mortality rates from three largest causes, Norway 2015. Source: OECD

**Fig. 2:**
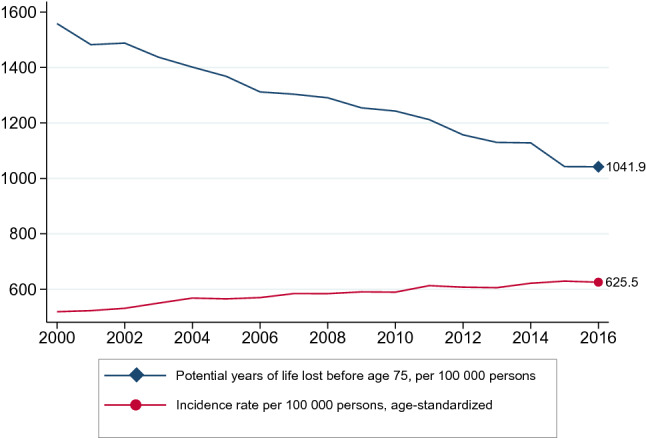
Cancer incidence rate and premature (before age 75) cancer mortality rate, Norway 2000–2016. Source: OECD and Norwegian Institute of Public Health

Worldwide, cancer is the second largest cause of death after heart disease [32]. In Norway, cancer (malignant neoplasm) was the leading cause of premature mortality in 2015, and the number of life-years lost by cancer was 13% higher than that by external causes and 157% larger than by diseases of the circulatory system (Fig. 1). Nevertheless, the premature cancer mortality rate has been decreasing since 2000, while the incidence rate (number of new cases per 100,000 persons) has been increasing, as shown in Fig. 2. In 2016, there were 625.5 new cancer cases per 100,000 persons.

**Fig. 3:**
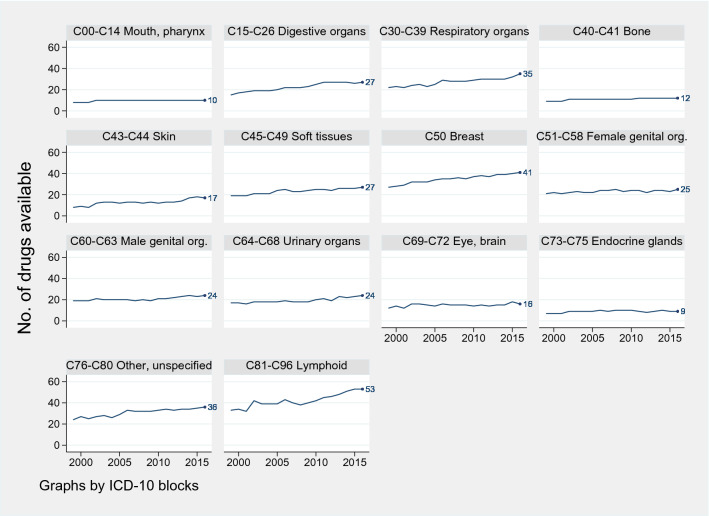
Number of drugs available to treat cancer, per cancer group, Norway 1999–2015. Drugs with at least 200 packages sold in a year

Cancer care in Norway is provided universally by the public healthcare system, and almost free of charge. Moreover, cancer is becoming more survivable, with some cancers becoming more so than others. This improvement may have pharmaceutical innovation as a contributor, as some cancers have seen more new drugs approved than others over the years. As shown in Fig. 3, the number of available drugs has been mostly increasing for the cancer groups C15–C26 digestive organs, C30–C39 respiratory organs, C43–C44 skin, C45–C49 connective and soft tissue, C50 breast, C64–C68 urinary organs, and C81–C96 lymphoid/hematopoietic tissue with the greatest increase. The remaining cancer groups have had a stable number of treatments available over this period.

Cancer treatment is complex, and a large number of approved drugs may matter. First, the treatment of one cancer patient can include radiotherapy, chemotherapy, antibodies, and molecules (in addition to surgery in some solid tumors). Second, cells in most cancers accumulate new mutations as they grow, and some of these mutations can make the cells resistant to chemotherapy and molecules. Thus, cancer drugs are often used in combination, since there is less resistance to two or three drugs at the same time [32]. Third, treatment may change over time: one drug may work well for some time until evidence of resistance, then a new drug is given to the patient until it also stops working, and so on until the end of the line of approved drugs. Nevertheless, the physician’s preference and the persistence of past practices may play a role in pharmaceutical demand, and in some cases, older drugs may be prescribed even if there are newer ones available [1]. Additionally, the diffusion of new drugs may be low due to uncertainties about their efficacy and safety.

**Fig. 4:**
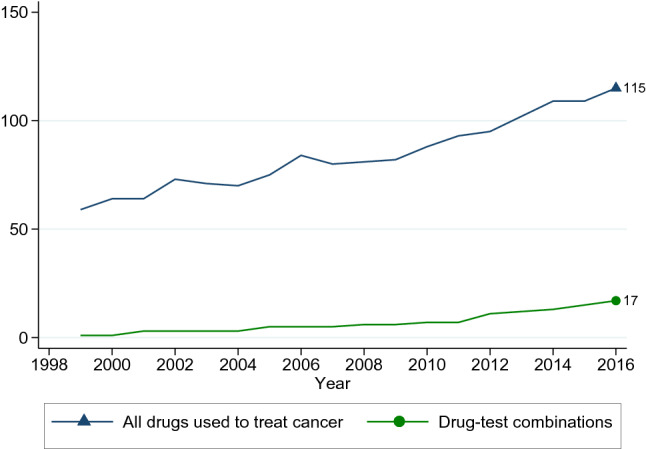
Number of drugs available to treat cancer during 1999–2016 in Norway. Drugs with at least 200 packages sold in a year

The improvement in health for some cancer types may also be related to the development of biomarker technology. Cancer drugs work in different ways, and biomarker tests have allowed for a better understanding of the disease and whether some drugs are more appropriate against a cancer with a specific set of mutations in comparison to others. As shown in Fig. 4, the number of pharmaceutical products (all drugs) available to treat cancer in Norway in 2016 is almost double the number in 1999. The number of “drugs that require testing for at least one predictive biomarker” (drug-test combinations or biomarker-guided therapies) went from zero in 1999 to 17 in 2016, with the greatest increase from 2012 onwards.

Currently, no health technology assessment guideline separately exists for cancer biomarkers or biomarker-guided therapies in Norway. They are assessed under the same guidelines as other nonguided therapies. In Norway, two approvals are required for new health technologies to access the Norwegian market; regulatory approval from the European Medicine Agency (EMA) and reimbursement approval from the Norwegian Medicine Agency (NoMA). NoMA collects relevant information for the assessment and conducts the assessment (e.g., Single Technology Assessment). Some information is often kept confidential and not presented in the public domain such as the discounted price of the drug.

## Data

Our analysis is based on patient-level data from the Cancer Registry of Norway and on drug sales data from the Norwegian Drug Wholesales Statistics (Grossistbasert Legemiddelstatistikk), Norwegian Institute of Public Health. The Cancer Registry of Norway contains information about each patient’s date of diagnosis, age at the time of diagnosis, county of residence, gender, cancer type (tumor location) indicated according to the WHO ICD-10 codes, and stage at diagnosis (local, regional, distant or unknown spread). The Cancer Registry also contains vital status information, date of death, and cause of death if the cause was cancer.

### Pharmaceutical data

Data on cancer drugs and the cancer type that each drug is indicated to treat (specified according to the WHO ICD-10 codes) were obtained from Thériaque, a drug database that contains official information on drug indications. The cancer types are the 47 malignant neoplasm ICD-10 codes (see appendix, Table 5 for a description of the codes).

We use data on drug sales from the wholesaler-based drug statistics database as a proxy for drug utilization at the national level. This database contains monthly total drug sales in number of packages, amount of active ingredient (e.g., kilograms), and the pharmacy purchase price from the wholesaler for the period between 1999 and 2016. For our main analysis, we assume that a drug was used/available in a given year if at least 200 packages were sold that year.^2^ Drug availability is not the theoretically ideal measure, since health outcomes are more strongly related to drugs actually used to treat the patient than drugs that could be used [18]. A preferable measure is, therefore, the exact drug consumption by each patient, informing us of who consumed which drug and when.^3^ However, these data are not available in a central registry, and hence, we use the variation in the number of drugs available (*Drugs*) as an indicator of the “stock of ideas” previously developed.

### Biomarker data

Information on drugs labeled with biomarker requirements can be found at the FDA website and at PharmGKB. These databases present information on which drugs require testing for biomarker(s) before prescription, and the cancer type they are meant to treat. Some drugs require more than one biomarker test (for instance, both KRAS and EGFR tests are required before the prescription of cetuximab for colorectal cancer), and some biomarker tests are used for more than one drug (for instance, the HER2 test is required before prescription of trastuzumab and lapatinib for breast cancer).

The exact official date of introduction of the biomarker tests in Norway is unavailable.^4^ Thus, we approximate the dates by obtaining information on the year of introduction of each biomarker test from oncologists and pathologists affiliated with the Centre for Cancer Biomarkers (CCBIO) at the university hospital in Bergen, Norway. They also confirmed the drugs that require a predictive biomarker test (or companion biomarker) before prescription and their corresponding biomarker (see appendix, Table 6).

**Fig. 5:**
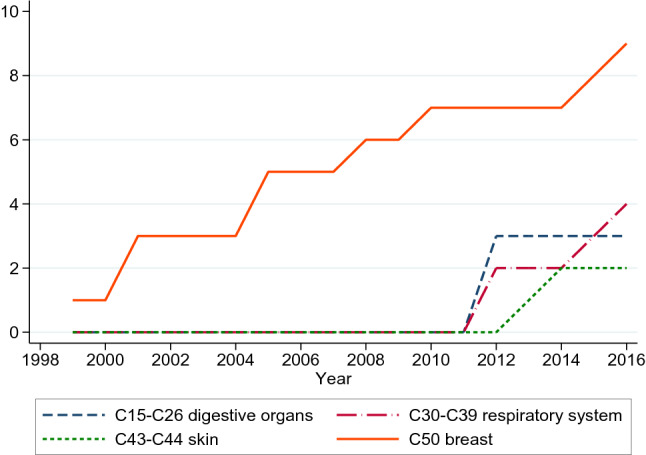
Number of drugs that require biomarker testing for treating four cancer groups, 1999–2016 in Norway. Drugs with at least 200 packages sold in a year

In this paper, we analyze the effect of biomarker testing to guide treatment on health outcomes in Norway during the period 1999–2016, controlling for the total number of cancer drugs available (*Drugs*). We further distinguish the type of drugs into therapies that require biomarker testing before prescription (*Guided*) from those that do not (*Nonguided*). As Table 6 and Fig. 5 indicate, the first biomarker-guided therapies were used for the treatment of breast cancer, testing for estrogen and progesterone receptors and HER2. Since 2012, other biomarker-guided therapies emerged for the treatment of cancer in digestive organs (especially relevant for colorectal cancer with EGFR and KRAS testing, and gastric cancer with HER2), skin cancer (melanoma with BRAF and NRAS), and cancer in the respiratory system (lung cancer with EGFR). We use this variation to assess whether patients with access to at least one biomarker test (*Biomarker*) had greater improvement in health outcomes.

### Health outcomes

Premature mortality is our first measure of health outcome. Specifically, it is measured as *potential years of life lost before age 75 or 65 (PYLL75 or PYLL65)*.^5^ This is calculated by subtracting the age at the time of death from 75 or 65 and expressed as a logarithm (*logPYLL*75, *logPYLL*65). In this case, we use a dataset of individuals who died with cancer in the period 2000–2016. Premature mortality allows us to look at the effect of biomarker testing and drugs that were available the year before death. Although we do not have data on which drugs were given to a patient nor when, it is uncommon for new drugs to be prescribed as initial treatment (first-line treatment). Hence, the advantage of using premature mortality as the outcome is that it captures the effect of new drugs even if they are prescribed at the end of the treatment period. As mentioned in Sect. 2, medical doctors usually prescribe older therapies first as they are more aware of their efficacy and safety (there may be unrecognized adverse drug reactions), while newer drugs are usually prescribed after unsuccessful treatment with previously launched drugs.

**Fig. 6:**
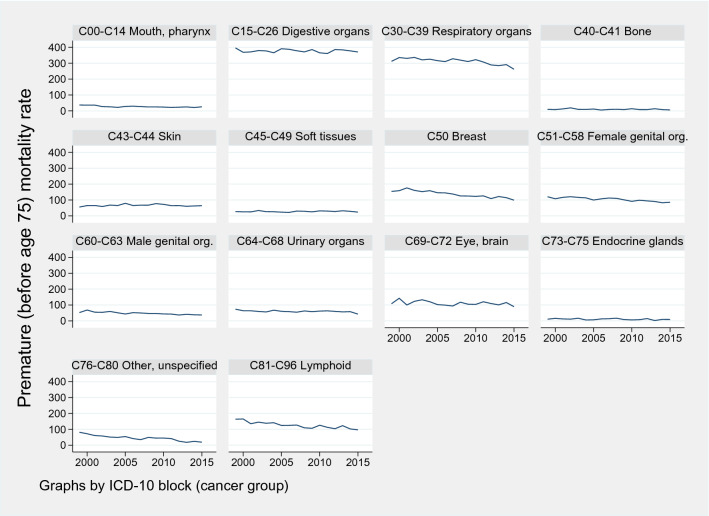
Years of potential life lost at age 75 per 100,000 persons below age 75, per cancer group, Norway 1999–2015

Figure 6 documents the evolution of the potential years of life lost before age 75 per cancer group. The potential years of life lost have decreased slightly over the period 1999–2016. This decrease in premature mortality is more visible for respiratory system cancers (C30–C39), breast cancer (C50), and cancers of lymphoid/hematopoietic tissue (C91–C96). However, this drop in premature mortality cannot be explained by a decline in cancer incidence (number of new cases per year), since incidence rates before age 75 have been increasing for most cancer groups, as shown in Fig. 8 in the appendix. It can, however, be due to an improvement of treatment.

**Fig. 7:**
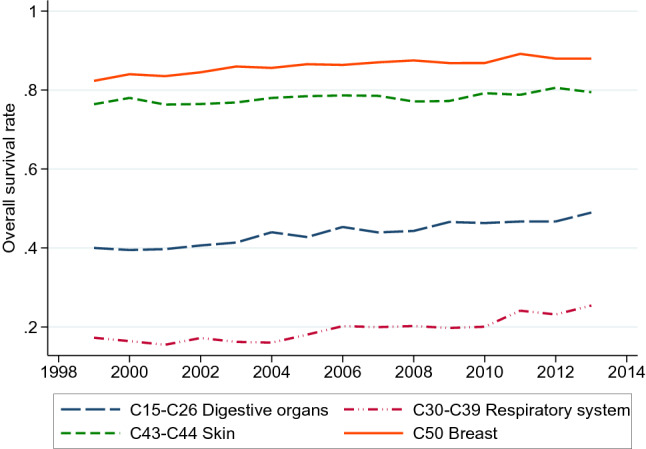
Overall survival rate for four cancer groups, Norway 1999–2013

**Fig. 8:**
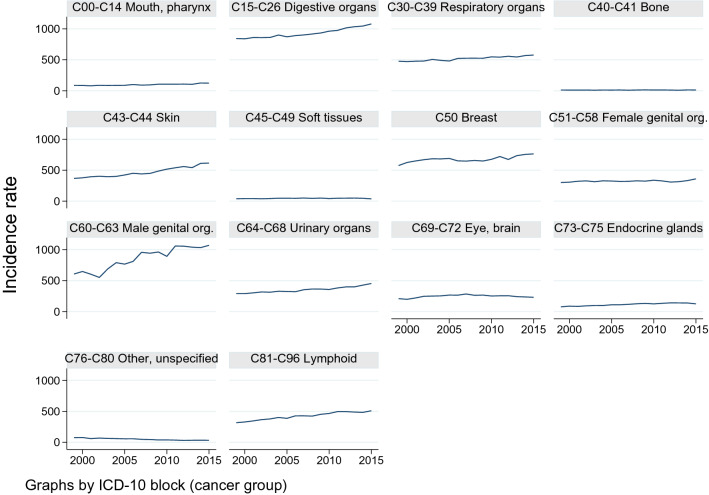
Incidence rate before age 75, per cancer group, Norway 1999–2015

Another outcome we analyze is the *3-year survival*. Since our dataset follows patients until 2016, the analysis of 3-year survival will only look at patients diagnosed in the period 2000–2013. As Fig. 7 indicates, the overall 3-year survival rate for the four cancer groups in which most biomarker tests were introduced seems to have had a small increase over this period. Five-year survival has been commonly used as a health outcome for the study of cancer patients (for example, [8]); however, most of the biomarker tests were introduced in Norway in 2012, and given the years of data we have available, studying the 5-year survival would exclude their effect. Therefore, we focus on the effect of cancer biomarkers and drugs available the year before diagnosis on the probability of being alive three years after diagnosis. When interpreting the effects on subsequent 3-year survival, however, it is important to keep in mind that this measure may mostly capture the effect of the initial treatment, which may not be related to a biomarker test or a new biomarker-guided therapy.

### Sample

The final sample for the analysis on premature mortality differs from the sample for the analysis on survival. In the premature mortality analysis, the sample consists of individuals who died due to cancer between 2000 and 2016, including those who were diagnosed any time before 2000. When analyzing the effect of drugs and biomarkers on the probability of surviving 3 years after diagnosis, we use a sample of individuals diagnosed between 2000 and 2013.

In both samples, we impose similar restrictions. The samples of cancer patients are restricted to individuals who were aged between 30 and 75 years old when they were diagnosed with cancer. We excluded patients younger than 30 years old, since cancer in children, adolescents, and young adults has a different biology from older patients, and treatment may vary as well [3]. Additionally, we exclude patients older than 75 years, to reduce unobserved comorbidities that may affect treatment and survival, which are more likely to occur in this age group.

Furthermore, approximately 22% of the patients in the premature mortality sample and approximately 13% of the individuals in the remaining survival sample were diagnosed with more than one type of cancer. For those individuals, there may be more drugs available depending on how many different diagnoses they had and on the type of cancers they were diagnosed with. To avoid further medical complexity of treatments, we exclude those individuals and restrict the analysis to patients diagnosed with cancer only once (or for the first time). Table 1 reports the summary statistics of the samples used in the premature mortality analysis and in the analysis on 3-year survival.^6^

## Empirical strategy

We base our analysis on the idea that our model is a health production function, where the health outcomes depend on the stock of approved treatments. Cancer-type fixed effects are included to take into account unobserved characteristics and time-invariant variables specific to the type of cancer (for instance, consistently higher survival probability for some cancer types than others). Moreover, time fixed effects are included to control for common shocks and a potential trend in the data (for instance, an overall decline in premature cancer mortality, or a common increase in survival probability across cancer types). We specify our first regression model as follows:

1 $$\begin{aligned} \begin{aligned} Y_{{{\text {ist}}}}&= \beta _{0} + \beta _{1} {\text {Biomarker}}_{{{\text {st}} - 1}} + \beta _{2} {\text {Drugs}}_{{{\text {st}} - 1}} \\&\quad + \beta _{3} \left( {{\text {Biomarker}}_{{{\text {st}} - 1}} \times {\text {Drugs}}_{{{\text {st}} - 1}} } \right) \\&\quad + \delta _{1} X_{i} + \delta _{2} {\text {Prevalence}}_{{{\text {st}}}} + \theta _{s} + \eta _{t} + \varepsilon _{{{\text {ist}}}} , \\ \end{aligned} \end{aligned}$$

where *i* defines each individual in the sample, *s* is the cancer type of the individual; $\theta$ represents the cancer-type specific effect to control for stable between-type differences, $\eta$ is the year of diagnosis fixed effect to control for aggregate shocks and trends, and $\varepsilon$ is an error term. $Y_{{{\text {ist}}}}$ represents *logPYLL*75, *logPYLL*65, or $\textit{3-year survival}$. Moreover, *logPYLL*75 and *logPYLL*65 are the logarithmic transformation of the number of years of life lost before age 75 and 65 because patient *i* died due to cancer type *s* in year *t*, and $\textit{3-year survival}$ is a dummy variable that equals one if patient *i* with cancer type *s* is still alive 3 years after being diagnosed. The baseline strategy in model (1) is, thus, to regress health outcomes onto a dummy variable that is equal to one if at least one predictive biomarker test was available (${\text {Biomarker}}_{{{\text {st}} - 1}}$),^7^ the number of drugs available (${\text {Drugs}}_{{{\text {st}} - 1}}$) to treat the cancer type *s* that patient *i* is diagnosed with the year before death (if $Y_{{{\text {ist}}}}$ is *logPYLL*75, *logPYLL*65) or diagnosis (if $Y_{{{\text {ist}}}}$ is *3-year survival*), and the interaction between the two variables (${\text {Biomarker}}_{{{\text {st}} - 1}}\times {\text {Drugs}}_{{{\text {st}} - 1}}$). $X_{i}$ is a vector of covariates to control for patient characteristics, including age at diagnosis, gender, stage, and county of residence at diagnosis. Since it is likely that pharmaceutical firms are more interested in developing drugs and biomarker tests for diseases with larger numbers of patients, we also control for cancer *Prevalence* (transformed into a logarithmic variable), here defined as the number of persons who have been diagnosed with cancer type *s* within the previous 10 years who are still alive at the end of year *t*.^8^ Furthermore, since it may take some time until new drugs begin to be widely prescribed (doctors may not be familiar with the efficacy and safety of a new drug), we consider the effect of *Drugs* and *Biomarker* testing already available the year before the patient died (when premature mortality is the outcome) or was diagnosed (when survival is the outcome).

The main coefficients of interest in Eq. (1) are $\beta _{1}$, the main biomarker testing effect, and $\beta _{3}$ from the interaction between testing for biomarkers and the number of cancer drugs available. This interaction term provides an estimate of the additional effect of drugs given that predictive biomarkers are available, or the additional effect of biomarker testing given the number of drugs available. We test for this synergy effect since the results from biomarker tests on patients help define the treatment process, such that those identified by the test as “responders” take a biomarker-guided therapy that is more effective for them, while the “nonresponders” can benefit more from other drugs and avoid the adverse events from the guided therapy.

An alternative way to analyze whether biomarker testing is associated with health outcomes is to separate the effect of drugs that require biomarker testing from those that do not. We address this by estimating the following equation:

2 $$\begin{aligned} \begin{aligned} Y_{{{\text {ist}}}}&= \beta _{0} + \beta _{1} {\text {Guided}}_{{{\text {st}} - 1}} + \beta _{2} {\text {Nonguided}}_{{{\text {st}} - 1}} + \delta _{1} X_{i} \\&\quad + \delta _{2} {\text {Prevalence}}_{{{\text {st}}}} + \theta _{s} + \eta _{t} + \varepsilon _{{{\text {ist}}}} . \\ \end{aligned} \end{aligned}$$

In this way, we test whether premature mortality (*logPYLL*75, *logPYLL*65) or survival probability (*3-year survival*) depends on the number of therapies that require biomarker testing (${\text {Guided}}_{{{\text {st}} - 1}}$) and on the number of those that do not (${\text {Nonguided}}_{{{\text {st}} - 1}}$), given the availability of therapies to treat cancer type *s* that patient *i* is diagnosed with in the year before death or diagnosis. Biomarker-guided therapies can not only be targeted to particular types of patients who would otherwise not have an effective treatment available but can also disseminate the practice of biomarker testing, consequently avoiding adverse reactions in potential nonresponders who ultimately do not consume these guided drugs.

The standard errors in all specifications are clustered within 47 cancer diagnoses (as provided by the Cancer Registry of Norway).

## Results

### Model 1: Biomarker testing and total number of drugs

Table 2 presents the results from estimating Eq. (1) and, for comparison, a regression without the interaction term using premature mortality (potential years of life lost before age 75 and 65) as the dependent variable in the first four columns, and survival 3 years after diagnosis as the dependent variable in the last two columns.^9^ All specifications exploit the variation with cancer-type fixed effects and year of diagnosis fixed effects. The effects of patient characteristics controlled for are not reported, but they generally have statistically significant coefficients. We are mainly focused on the relationship between the use of biomarker tests and health outcomes.

In the first four columns, the estimates of the biomarker main effect (*Biomarker(s) available*) are statistically significant and their sign indicates a reduction in premature mortality in all specifications: the coefficient is negative and statistically significant at the 1% level. Additionally, the coefficient of *Drugs* is negative and statistically significant, which is in line with the initial hypothesis suggesting that with a higher “stock” of drugs approved, the level of premature mortality tends to decrease.

In columns (1) and (3), the interaction term ($Biomarker(s)\times Drugs$) that represents the additional effect of drugs due to biomarker testing on premature mortality is positive, as opposed to the *Biomarker*(*s*) main effect, but it is not statistically significant. Moreover, the average marginal effects of testing for a biomarker to predict drug response on *logPYLL*75 and *logPYLL*65 are similar: testing for biomarkers is associated with an average decrease of premature mortality before age 75 and 65 by 14.9% and 19.2%, respectively.^10^

Regarding the effect on the probability of being alive 3 years after diagnosis in column (5), *Biomarker*(*s*) has a positive and statistically significant coefficient, while the interaction term has a negative and statistically significant coefficient, suggesting that the total effect of biomarkers available on survival probability decreases as the number of drugs available increases. However, the average marginal effect of biomarkers available on 3-year survival is positive but not statistically significant. In contrast, when the interaction term is excluded (column (6)), the coefficient of biomarker availability is not statistically significant.

The negative estimate of the interaction between biomarker testing and the number of drugs on survival may at first sight be paradoxical. One may expect that as more biomarkers tests and drugs are available for cancer treatment, the synergy between them might be positive since more patients with different tumor characteristics should benefit. However, we find that their interaction has a negative association with survival. Implications of this finding will be considered in the discussion section.

### Model 2: Guided vs. nonguided therapies

By estimating Eq. (2), we further separate the effect of the number of cancer drugs that until the end of 2016 had not required a biomarker test to be performed before prescription (*Nonguided* drugs) from the number of cancer drugs that at least at some point in the period of the study require biomarker testing (*Guided* drugs). Table 3 reports the results using *logPYLL*75, *logPYLL*65, and *3-year survival* as dependent variables.

A negative and statistically significant relationship is depicted between *Guided* therapies and premature mortality in the first two columns. Hence, one additional biomarker-guided therapy is associated with a 4.1% decrease in potential years of life lost before age 75 and a 5.6% decrease in potential years of life lost before age 65. In this case though, while the coefficient for *Nonguided* therapies is negative, it is not statistically significant. Additionally, the difference between the coefficients on *Guided* and *Nonguided* drugs is statistically significant, so the two types of drugs had different effects on premature mortality. This suggests that the reduction in premature mortality is associated with guided therapies rather than nonguided therapies. More specifically, it confirms the inverse relationship between biomarker(s) testing and premature mortality. This can be linked to the high response rate to these drugs that target a specific group of patients, and/or the practice of biomarker testing that becomes more common as more guided therapies are introduced in the market and can avoid adverse reactions in other patients who do not benefit from them.

Interestingly, in the last column, a positive relationship is depicted between *Nonguided* therapies and *3-year survival*, while the coefficient for *Guided* therapies is not statistically significant. Given this, we do not find an association between biomarker testing and 3-year survival in this model, but we find that one additional nonguided therapy available is associated with an increase of 0.8 percentage points in the probability of surviving 3 years after diagnosis.

Overall, the results suggest that biomarker-guided therapies are associated with a reduction in premature mortality, while nonguided therapies are associated with an increase in survival probability. Potential mechanisms behind this result are mentioned in the discussion section.

## Robustness checks

This section presents additional results to investigate the robustness of the main findings. The most relevant checks of the model in Eq. (1) are reported in the appendix. Estimates from robustness checks of the model in Eq. (2) do not differ in a remarkable way and are available upon request.

### Alternative specification choices

One concern when working with drug sales data is to know how much of a pharmaceutical product must be sold to make a visible impact on health. The original data show that several new drugs sales increased gradually, selling a very low number of packages during the first months (sometimes below 10 packages), or with long breaks between months. To avoid drugs used in clinical trial phases or not yet known by the majority of physicians, while including some important and very recent biomarker-guided therapies in the sample, we needed criteria for when the drug should be considered in the analysis.^11^ In the main specifications, the criterion was 200 or more packages sold in a year. As a robustness check, we also perform the main analysis with different numbers of packages sold (50, 100, and 500). The results using 200 packages sold as the determinant for whether the drug was available are similar to those using 50, 100, or 500 packages. These results are reported in the appendix, Table 8.

As an alternative to the linear probability model for the analysis on survival, we estimated a logistic regression model, which has the advantage of restricting the predicted probability to be between 0 and 1. Table 9 in the appendix presents the results from estimating Eq. (1) with logistic regression. The average marginal effect suggests an average increase of the probability of surviving 3 years after diagnosis by 1.4 percentage points associated with biomarker testing being available. However, the implementation of fixed effects in the logit model may lead to inconsistent estimators [11]. Hence, we prefer the linear model in the main specification.

Additionally, we restrict the main analysis to include only individuals with cancer types that at least at some point in time have biomarker tests available in the treatment process. This includes gastric (C15, C16), colorectal (C18, C19–20, C21), lung (C33–C34), melanoma (C43), breast (C50), and lymphoma (C81) which in total consist of 9 ICD-10 cancer types. The results are reported in Table 10 in the appendix, and the premature mortality specifications confirm the baseline results with respect to biomarker testing and guided therapies, but the 3-year survival specifications differ from the baseline results, as we do not find statistically significant estimates for biomarker testing. However, this analysis may remove considerable variation since all cancer types except lymphoma were using biomarker tests by 2012.

Moreover, we estimate the main model without controlling for individual characteristics and prevalence. We also estimate the model with age at diagnosis as the only covariate. As shown by the *R*-squared in Table 11 in the appendix, age is an important control variable when the outcomes are potential years of life lost before age 75 and 65. The exclusion of most controls mainly increases the estimates of the impact of the number of drugs available compared to the main model, while the biomarker estimates do not change much.

One may argue that the biomarker tests on their own should not have an effect, but the effect of drugs may change with testing. Biomarker testing is used to let physicians know whether some drugs can be used for treatment so that the right drug is given and adverse events are avoided. Therefore, we also estimate the model by removing the main effect of biomarker tests and focusing only on the interaction term (Table 12 in the appendix). Although biomarker testing appears to have decreased the effect of all drugs and guided therapies on survival, which does not correspond to the main results, the results on premature mortality appear to be qualitatively robust. Indeed, the effect of drugs on premature mortality is greater when biomarker testing is available, suggesting that patients benefit more from it.

Additionally, as a robustness check, the premature mortality models are estimated with the levels, rather than logarithm, of potential years of life lost. The results do not qualitatively differ in a remarkable way from the main specification (available upon request).

### Heterogeneity

A large fraction of the cancer patients are aged above 75 at the time of diagnosis, and patients with comorbidities may be too fragile for more invasive treatment such as surgery and instead benefit more from cancer drugs and biomarker tests. Hence, we check the robustness of our main results by estimating the model by setting the age limit at the life expectancy in Norway, which was approximately 82 years in 2013. To perform the robustness checks, we limit the sample to three groups: patients aged 30–82 years, 30–50 years, and 50–82 years, as most screening programs are recommended from age 50 (for example, the Norwegian Breast Cancer Screening Program targets women aged 50–69). The results are shown in the appendix, Table 13. The estimates based on patients aged 30–82 and 50–82 years are very similar to the estimates based on the age threshold of 75 and 65 years in the main analysis. However, we find small differences in the sample aged 30–50 years: the estimates of the number drugs available are not statistically significant, and the interaction term has a positive association with premature mortality before age 50. Overall, the biomarker effect corresponds to our main findings. Nevertheless, coefficients are not significantly different across age groups

Finally, we estimate the main models of premature mortality and survival in subgroups of patients (restricting the sample to patients with distant spread and with localized spread in separate regressions), since the tumor characteristic varies for the majority of the individuals in each specification. A large fraction of the patients included in the sample of the main analysis on premature mortality have a tumor with a distant spread at diagnosis, while for the survival analysis sample, the cancer stage with the largest fraction of patients is localized spread. The results are shown in the appendix, Table 14. The results on premature mortality are qualitatively similar to the main results, and spread does not seem to matter much for the health effect of treatment (the coefficients for distant and localized spread are not significantly different from each other). Note, however, that the data are noisy for the models on survival of patients with distant and localized spread (*R*-square of 0.026 and 0.031, respectively). This may be partly explained by the unobserved timing of treatment, as this model estimates the effect of treatment at the time of diagnosis and most new drugs may be given later on. Additionally, the cancer stage may change, but that is not reported in our data.

Overall, the results on premature mortality are more robust than the results on survival. Further potential reasons for this finding are pointed out in the discussion section.

## Discussion

### Mechanisms

The results reported in the previous sections indicate a negative association between the biomarker and drug interaction term and the improvement of health, even though one could expect that the synergy between testing for biomarkers and the availability of more cancer drugs should improve health outcomes. It is not obvious why we find this paradoxical effect of the interaction term on survival and on premature mortality in some specifications. A potential reason is the time it takes to test and obtain the results of the biomarker, which is a disincentive for medical doctors to prescribe biomarker-guided drugs. Instead, oncologists can prescribe nonguided therapies to speed up the treatment process. They may prefer to do so because nonguided therapies can provide a similar treatment to cancer sufferers without the hassle of testing them first [6].^12^ Another possible explanation for the unexpected sign of the interaction term is that the doctors’ persistence of past practices and preference for older drugs in the market [1] plays a crucial role in determining the type of drugs actually used in clinical practice: doctors may stick to few biomarker-guided therapies instead of using all of those available. When the first biomarker-guided drugs are launched into the market, they are prescribed to a specific group of patients who are biomarker positive.^13^ Other drugs using the same biomarker test for the same type of cancer can be introduced later on, but physicians may be more familiar with the earlier biomarker-guided drugs and prescribe them instead of the newer ones, although doctors may change to the new guided-therapy more easily with assurance when its companion tests are approved to be clinically effective and cost-effective [12, 30]. As a consequence, the new guided therapies may not be prescribed to as many patients as the first biomarker-guided therapies. Those patients could be deprived of potentially beneficial drugs that target their specific type of cancer better or have an incremental improvement in health relative to earlier drugs. Hence, the total effect of biomarker testing decreases as the number of this type of drug increases. Presumably, the health system becomes increasingly complex and all of the reasons described above are associated with a reduction in the effect of cancer biomarkers as the number of drugs available increases. In general, more testing leads to more diagnoses, and each diagnosis requires more attention. However, more diagnoses and more treatment options increase the complexity of healthcare, and it is challenging for oncologists to prioritize correctly [7]. In other words, as the available number of drugs increases, the complexity of treatment decisions increases, and the more difficult it becomes to match the right patient to the right drug.

Our results from model (2), point out that the reduction in premature mortality is associated with guided therapies rather than nonguided therapies, while biomarker-guided therapies do not have a statistically significant effect on the probability of surviving 3 years after diagnosis. This can be attributed to differences in the samples for the regression on premature mortality and on survival and that it is plausible that cancer patients at the end of life benefit more from new drugs compared to patients who have just been diagnosed. Since biomarker-guided drugs are prescribed to relatively few patients (only those who are identified as likely to respond by the biomarker test) and their introduction into clinical practice has been very recent, doctors are not so familiar with these drugs and are not confident about their safety and efficacy. Hence, doctors do not prescribe biomarker-guided therapies to every patient but perhaps to those who are at an advanced stage of cancer and have a poor prognosis, or who have tried other nonguided drugs without any success. This can prolong life for those who will nevertheless die of cancer, which is the case of the sample used to analyze premature mortality. On the other hand, nonguided drugs have been used for a longer period in cancer treatment and used more as first-line therapy (first treatment given for a disease), and the analysis on 3-year survival may capture mostly the beginning of the treatment. Thus, this can explain why only the use of nonguided therapies is associated with an increase in the probability of survival 3 years after diagnosis. Furthermore, new biomarker-associated drugs are often tested in the metastatic setting and then move into the adjuvant setting where a greater impact on survival could be achieved as we could observe in targeted therapies of melanoma [4, 29]. In other words, new guided therapies are more likely to be used in later lines of therapy where we see progressively less positive impact on patient survival.

In contrast to our survival results, we find a more consistent relationship between premature mortality and biomarker tests and cancer drugs. The results suggest a decrease in premature mortality before age 75 and 65 associated with the availability of biomarker testing and drugs. There are some explanations for why that relationship is more consistent when we evaluate premature mortality than when we analyze survival probability. First, the potential benefit of treatment is not as high for people whose prognosis is good as it is for people with a poorer prognosis, but they are still at risk of drug adverse events [7]. With overtreatment, that risk can exceed the benefit. The sample used to analyze the relationship between survival and biomarkers may consist of a large number of patients who were diagnosed early enough to have a good prognosis and could be at risk of overtreatment. On the other hand, the sample used to make the analysis on premature mortality consists of patients who died due to cancer, and we look at the end of their lives, when prognosis might have been poorer than at diagnosis.^14^ Another possibility is that the newest drugs are often not used as first-line treatments, and patients are not consuming them right after receiving their diagnosis. Such drugs are often prescribed after unsuccessful treatment with earlier therapies. When survival is the outcome, we are looking only at the effect of treatment at the time of diagnosis (not necessarily when biomarker-guided therapies are used), while using premature mortality can capture more of the effect of therapies used during a longer or later period of treatment. It can take years for new therapies to obtain the approval to be used as the first treatment given. Perhaps in the future, it may be possible to study the effect of biomarker tests and guided therapies on survival with stronger robustness.

### Potential cost per life-year gained

The estimates of the effect of biomarker-guided drugs on premature mortality from Table 3 can be used to calculate a rough estimate of the life-years gained in 2016 from biomarker-guided drugs. For example, knowing that for cancer types where biomarker testing was introduced, the total premature mortality before age 75 decreased from 29,519 years of life lost in 2000 to 24,603 years of life lost in 2016 (before age 65 decreased from 12,198 in 2000 to 8282 in 2016), we can calculate how many of the 4916 years (3916 years) that decreased during the period 2000–2016 are associated with the increase in the number of biomarker-guided therapies. These calculations (for patients before ages 75 and 65) are shown in Table 4. Our approach is similar to the one used in Lichtenberg [18]. The mean 2000–2015 increase in the number of guided drugs across the cancer types where biomarker testing was implemented was 2.9. The negative sign of the estimates of $\beta _{1}$ implies that premature mortality would have been higher in the absence of biomarker-guided therapies. The percentage change in premature mortality associated with the increase in the number of guided drugs is given by $[\exp ( - \beta _{1} \times \Delta {\text {Guided}}) - 1] \times 100$. For example, premature mortality before age 75 in 2016 would have been 13% higher if no guided drugs were implemented: it would have been 27,727 rather than its actual value of 24,603. As shown in line 4 of Table 4, this implies that 3124 life-years before age 75 were gained in 2016 linked to the availability of biomarker-guided therapies. The estimate of the number of life-years before age 65 gained is, thus, 1470.

On the other hand, 2016 pharmaceutical expenditure would presumably have been lower in the absence of guided therapies. Line 5 of Table 4 shows estimates of expenditure in 2016 on biomarker-guided therapies used to treat cancer that became available in Norway during the period 2000–2015. Data on sales in terms of purchase price from the Norwegian Drug Wholesales Statistics were used to make a rough estimate of the 2015 expenditure on guided drugs. These data indicate total sales from the wholesalers on each drug (by generic name) used by patients of all ages. Data on total prevalence in cancers with guided therapies available indicate that 64% of the patients are aged between 30 and 75, and 34% are aged between 30 and 65. Thus, we assume that 64% of 2015 expenditure on guided drugs to treat cancer was for patients below age 75 and similarly, that 34% was for patients below age 65. This implies that the cost per life-year gained from biomarker-guided drugs was NOK 64,212 (EUR 6397) before age 75 and NOK 72,517 (EUR 7224) before age 65. In Norway, there is no explicit threshold at which an intervention is considered cost-effective. However, a threshold of EUR 30,000 per QALY (quality-adjusted life-year) was assumed in the economic evaluation of point-of-care C-reactive protein testing in Norwegian patients with lower respiratory tract infections by Oppong et al. [25]. Our estimates of cost per life-year gained before age 75 and 65 from guided drugs are well below this threshold.

A shortcoming of the cost per life-year analysis is that it was not possible to consider spending on the biomarker tests. This occurs because the coding systems of biomarker tests are not yet established in medical recording systems in Norway. Moreover, the sales in terms of purchase price from the wholesaler to pharmacies, hospitals, nursing homes and nonpharmacy outlets do not correspond to the final expenditure. The deviation from the real cost and the absence of the cost of biomarker testing in the analysis likely result in an underestimate of the cost per life-year from biomarker-guided therapies, and the sales in terms of purchase price can be seen as an estimated lower bound of expenditure on guided drugs.

### Limitations

Our results may capture an additional value of taking advantage of a treatment that can explain the high estimates of effects on premature mortality. Although premature mortality is used in the literature [16–18] to avoid lead-time bias that can affect survival, it may still not account for the positive externalities from older to newer treatments. A treatment that prolongs life will not only reduce premature mortality directly but also give patients the opportunity to benefit from newer drugs, since one drug may enable the patient to live long enough to use future treatments, which in turn may have better efficacy and safety [13]. This additional value is not disentangled in this work given the lack of data on individual drug consumption.

In addition to this, there are further limitations to acknowledge in this study. First, the data on the introduction time of biomarker tests to the Norwegian clinical practices were constructed based on expert opinions while referring to the regulatory year of relevant biomarker-guided therapies. Second, we assumed no differences in terms of ability to access new technologies in Norway. Given the universal healthcare systems in Norway, we assumed that all eligible patients would be able to access the new biomarker-guided therapies once the therapies are introduced in Norway. The results may differ from the ones found in this paper if, in contrast, patients are in healthcare systems with low insurance coverage and more likely to not consume some drugs due to high cost.

## Summary and conclusion

The biomarker technology to predict drug response has started being implemented in the treatment of cancer, but the adoption of biomarker testing in clinical practice has been slower than expected. The complexity of the science and imprecision of biomarker prediction are some of the factors that might have contributed to this phenomenon.

This study focuses on the analysis of biomarker testing’s health impact in the real world. Indeed, have cancer patients benefited from biomarker testing? We have studied the relationship between biomarker testing that predicts drug response and health outcomes, controlling for cancer drug availability, cancer types and patient characteristics, in a sample of cancer patients between 2000 and 2016.

The analysis has focused on three different health outcomes: premature mortality before age 75, premature mortality before age 65, and the probability of being alive 3 years after diagnosis. We find that the availability of these biomarker tests is associated with a decrease in premature mortality before age 75 and 65 and an increase in survival probability. We also find that the total effect of biomarker testing on survival decreases as the number of drugs available increases, suggesting that testing is more effective for cancer types with fewer treatments available. Furthermore, the main results of this analysis highlight the difference between treatments with biomarker testing and treatments without, i.e., the availability of biomarker-guided drugs reduces the potential years of life lost before age 75 and 65, but we do not find a consistent association between nonguided drugs (which do not require biomarker testing) and premature mortality. On the other hand, the number of nonguided therapies has a positive association with the probability of surviving 3 years after diagnosis, but we find a weaker statistical significance of the effect of biomarker-guided drugs on survival. We estimated that 3124 life-years before age 75 and 1470 life-years before age 65 were gained in 2016 associated with biomarker-guided drugs implemented in Norway during 2000–2015 and that the cost per life-year gained was NOK 64,212 before age 75 and NOK 72,517 before age 65.

The findings in this study shed light on the potential effects of biomarker tests that predict drug response. However, the availability of data does not permit a deeper analysis of the mechanisms. The benefits may vary based on the patient’s socioeconomic status, family history of disease, and biomarker results, and on the physicians’ decision-making. Furthermore, our study is likely to underestimate the effect of biomarker tests and guided therapies on survival, since these technologies are recent developments and the period in our analysis may not allow us to capture its long-term benefits. Additional biomarker tests and guided therapies have been approved and introduced in Norway after the time covered by our dataset. Future research should take advantage of a longer data series to better understand the complex relationship between health outcomes and biomarker-guided cancer treatment.

**Table 1:** Summary statistics of the variables used

	Premature mortality sample					Survival sample				
	Mean	St. dev	Min	Max	Sum	Mean	St. dev	Min	Max	Sum
Years of life lost before 75	11.62	8.964	0	45	879,769					
Years of life lost before 65	8.438	7.170	0	35	330,322					
Survival 3 years after diagnosis						0.704	0.457	0	1	129,687
Drugs available	13.91	9.853	0	46		13.30	10.70	0	43	
Biomarker tests available	0.281	0.625	0	2		0.273	0.636	0	2	
Age at diagnosis	60.64	9.568	30	75		59.87	10.64	30	75	
Gender dummy (1 = Female, 0 = Male)	0.458	0.498	0	1	34,710	0.488	0.500	0	1	89,948
Year of diagnosis	2005.7	5.920	1957	2016		2007.2	4.005	2000	2013	
Year of death	2007.9	4.928	2000	2016						
Prevalence	2833.1	3801.3	7	20,455		4332.8	4286.9	7	17,608	
Stage at diagnosis										
Localized	0.132	0.338	0	1	9994	0.396	0.489	0	1	72,998
Regional	0.225	0.418	0	1	17,053	0.213	0.410	0	1	39,313
Distant	0.438	0.496	0	1	33,140	0.182	0.386	0	1	33,575
Unknown	0.205	0.404	0	1	15,522	0.209	0.406	0	1	38,451
* No. of observations*	75,741					184,344

**Table 2:** Effect of testing for biomarkers on premature mortality and survival

	logPYLL75		logPYLL65		3-year survival	
	(1)	(2)	(3)	(4)	(5)	(6)
Biomarker(s) available	− 0.210*** (0.0724)	− 0.130*** (0.0386)	− 0.265*** (0.0935)	− 0.167*** (0.0522)	0.059** (0.0233)	− 0.028 (0.0187)
Biomarker(s) $\times$ drugs	0.004 (0.0034)		0.005 (0.0041)		− 0.004*** (0.0008)	
# Drugs available	− 0.021** (0.0091)	− 0.017** (0.0075)	− 0.025* (0.0133)	− 0.020* (0.0104)	0.008 (0.0024)	0.005 (0.0034)
Biomarker marginal effect	− 0.149*** (0.0399)		− 0.192*** (0.0546)		0.009 (0.0147)	
*R*-square	0.775	0.774	0.771	0.771	0.205	0.205
No. of obs	71,656	71,656	36,040	36,040	184,344	184,344

Ordinary least squares (OLS) coefficients with cancer-type fixed effects and year of diagnosis fixed effects. Standard errors in parentheses are heteroskedastic robust and corrected for clustering at the cancer type level. Controls include age at diagnosis, gender, stage, county of residence, and prevalence. Biomarker marginal effect reports the average marginal effect, where the effects are calculated for each observation and then averaged.

*$$p<0.1$$, **$$p<0.05$$, ***$$p<0.01$$

**Table 3:** Effect of biomarker-guided and nonguided therapies on premature mortality and survival

	logPYLL75	logPYLL65	3-year survival
Guided	− 0.041*** (0.0142)	− 0.056*** (0.0157)	− 0.006 (0.0080)
Non-guided	− 0.011 (0.0086)	− 0.011 (0.0128)	0.008*** (0.0028)
*R*-square	0.774	0.770	0.205
No. of obs	71,656	36,040	184,344

Ordinary least squares (OLS) coefficients with cancer-type fixed effects and year of diagnosis fixed effects. Standard errors in parentheses are heteroskedastic robust and corrected for clustering at the cancer-type level. Controls include age at diagnosis, gender, stage, county of residence, and prevalence.

*$$p<0.1$$, **$$p<0.05$$, ***$$p<0.01$$

**Table 4:** Calculation of cost per life-year gained in 2016 from biomarker-guided therapies

Line		Before age 75	Before age 65
1	2016 Premature mortality (PYLL)	24,603	8282
2	Increase in number of guided drugs 2000–2016 ($\Delta Guided$)	2.9	2.9
3	Guided drugs estimate ($\beta _{1}$)	− 0.041	− 0.056
4	Gain in life-years due to guided drugs: $[\exp ( - \beta _{1} \times \Delta {\text {Guided}}) - 1] \times 100)-1]\times PYLL$	3124	1470
5	Estimated expenditure in 2015 on guided drugs (*COST*)	NOK 200,600,000	NOK 106,600,000
6	Cost per life-year gained ($=COST/GAIN$)	NOK 64,212	NOK 72,517

## Appendix

See Tables 5, 6, 7, 8, 9, 10, 11, 12, 13 and 14 and Fig. 8.

**Table 5:** Cancer types by ICD-10 code

ICD-10	Cancer type
**C00**–**14**	**Mouth, pharynx**
C00	Lip
C01–02	Tongue
C03–06	Mouth, other
C07–08	Salivary glands
C09–14	Pharynx
**C15**–**26**	**Digestive organs**
C15	Oesophagus
C16	Stomach
C17	Small intestine
C18	Colon
C19–20	Rectum, rectosigmoid
C21	Anus
C22	Liver
C23–24	Gallbladder, bile ducts
C25	Pancreas
C26	Other digestive organs
**C30**–**34, C38**	**Respiratory organs**
C30–31	Nose, sinuses
C32	Larynx, epiglottis
C33–34	Lung, trachea
C38	Heart, mediastinum and pleura
**C40**–**41**	**Bone**
**C43**–**44**	**Skin**
C43	Melanoma of the skin
C44	Skin, non-melanoma
**C45**–**C49**	**Connective and soft tissue**
C45	Mesothelioma
C47	Autonomic nervous system
C48–49	Soft tissues
**C50**	**Breast**
**C51**–**58**	**Female genital organs**
C51–52, C57.7–9	Other female genital
C53	Cervix uteri
C54	Corpus uteri
C55	Uterus, other
C56, C57.0–4	Ovary etc.
C58	Placenta
**C60**–**63**	**Male genital organs**
C61	Prostate
C62	Testis
C60, C63	Other male genital
**C64**–**68**	**Urinary organs**
C64	Kidney (excl. renal pelvis)
C65–68	Urinary tract
**C69-72**	**Eye, brain and central nervous system**
C69	Eye
C70–72	Central nervous system
**C73-75**	**Endocrine glands**
C73	Thyroid gland
C37, C74–75	Other endocrine glands
**C39, C76, C80**	**Other or unspecified**
**C81**–**96**	**Lymphoid/haematopoietic tissue**
C81	Hodgkin lymphoma
C82–86, C96	Non-Hodgkin lymphoma
C88	Immunoproliferative disease
C90	Multiple myeloma
C91-95	Leukaemia

**Table 6:** Most used companion diagnostics for cancer in Norway

	Biomarker test	ESRPGR	HER2	HER2	KRAS	EGFR	EGFR	ALK	BRAF & NRAS	MSI	CD30
	Test indication	Breast	Breast	Gastric	Colorectal	Colorectal	Lung	Lung	Skin	Colorectal	Lymphoma
	Test date	1990	2005	2011	2012	2012	2012	2015	2012	2016	2015
ATC code	Drug generic name										
L01XC03	Trastuzumab	x	x	x							
L01XC06	Cetuximab				x	x					
L01XC08	Panitumumab				x	x					
L01XC13	Pertuzumab		x								
L01XC18	Pembrolizumab									x	
L01XE02	Gefitinib						x				
L01XE03	Erlotinib						x				
L01XE07	Lapatinib		x								
L01XE13	Afatinib						x				
L01XE15	Vemurafenib								x		
L01XE16	Crizotinib							x			
L01XE23	Dabrafenib								x		
L01XE25	Trametinib								x		
L01XE10	Everolimus	x									
L02BA03	Fulvestrant	x									
L02BG03	Anastrozole	x									
L01XE33	Palbociclib	x									
L01XC12	Brentuximab vedotin										x

**Table 7:** Summary statistics of the variables used (cancer types)

	Premature mortality sample					Survival sample				
	Mean	St. dev	Min	Max	Sum	Mean	St. dev	Min	Max	Sum
Cancer type (ICD-10)										
C00 Lip	0.000172	0.0131	0	1	13	0.00251	0.0501	0	1	463
C01–02 Tongue	0.00379	0.0614	0	1	287	0.00368	0.0606	0	1	679
C03–06 Mouth, other	0.00421	0.0648	0	1	319	0.00288	0.0536	0	1	531
C07–08 Salivary glands	0.00156	0.0394	0	1	118	0.00157	0.0396	0	1	290
C09–14 Pharynx	0.00727	0.0850	0	1	551	0.00623	0.0787	0	1	1148
C15 Oesophagus	0.0216	0.145	0	1	1633	0.00851	0.0919	0	1	1569
C16 Stomach	0.0346	0.183	0	1	2622	0.0177	0.132	0	1	3266
C17 Small intestine	0.00489	0.0697	0	1	370	0.00443	0.0664	0	1	816
C18 Colon	0.0898	0.286	0	1	6801	0.0714	0.257	0	1	13,157
C19–20 Rectum, rectosigmoid	0.0385	0.192	0	1	2919	0.0432	0.203	0	1	7956
C21 Anus	0.00156	0.0394	0	1	118	0.00248	0.0498	0	1	458
C22 Liver	0.0190	0.136	0	1	1438	0.00557	0.0744	0	1	1027
C23–24 Gallbladder, bile ducts	0.00693	0.0830	0	1	525	0.00565	0.0749	0	1	1041
C25 Pancreas	0.0678	0.251	0	1	5134	0.0253	0.157	0	1	4671
C26 Other digestive organs	0.00411	0.0639	0	1	311	0.00271	0.0520	0	1	499
C30–31 Nose, sinuses	0.00127	0.0356	0	1	96	0.00157	0.0396	0	1	290
C32 Larynx, epiglottis	0.00382	0.0617	0	1	289	0.00398	0.0630	0	1	734
C33–34 Lung, trachea	0.246	0.431	0	1	18,641	0.104	0.306	0	1	19,243
C37, C74–75 Other endocrine glands	0.00181	0.0425	0	1	137	0.0101	0.100	0	1	1864
C38 Heart, mediastinum and pleura	0.00131	0.0361	0	1	99	0.000656	0.0256	0	1	121
C39, C76, C80 Other or unspecified	0.0334	0.180	0	1	2527	0.0116	0.107	0	1	2130
C40–41 Bone	0.00191	0.0437	0	1	145	0.00151	0.0388	0	1	278
C43 Melanoma of the skin	0.0330	0.179	0	1	2498	0.0578	0.233	0	1	10,664
C44 Skin, non-melanoma	0.00136	0.0369	0	1	103	0.0241	0.153	0	1	4434
C45 Mesothelioma	0.00655	0.0807	0	1	496	0.00305	0.0552	0	1	563
C47 Autonomic nervous system	0.000198	0.0141	0	1	15	0.000271	0.0165	0	1	50
C48–49 Soft tissues	0.00759	0.0868	0	1	575	0.00608	0.0777	0	1	1120
C50 Breast	0.0687	0.253	0	1	5202	0.138	0.345	0	1	25,468
C51–52, C57.7–9 Other female genital	0.00189	0.0434	0	1	143	0.00304	0.0550	0	1	560
C53 Cervix uteri	0.0107	0.103	0	1	812	0.0159	0.125	0	1	2940
C54 Corpus uteri	0.00841	0.0913	0	1	637	0.0291	0.168	0	1	5366
C55 Uterus, other	0.00419	0.0646	0	1	317	0.000195	0.0140	0	1	36
C56, C57.0–4 Ovary etc.	0.0393	0.194	0	1	2974	0.0215	0.145	0	1	3967
C58 Placenta	0.0000264	0.00514	0	1	2	0.000114	0.0107	0	1	21
C61 Prostate	0.000845	0.0291	0	1	64	0.00143	0.0377	0	1	263
C62 Testis	0.0536	0.225	0	1	4061	0.157	0.364	0	1	28,918
C60, C63 Other male genital	0.000911	0.0302	0	1	69	0.0127	0.112	0	1	2350
C64 Kidney (excl. renal pelvis)	0.0246	0.155	0	1	1864	0.0230	0.150	0	1	4240
C65–68 Urinary tract	0.0201	0.140	0	1	1525	0.0337	0.180	0	1	6211
C69 Eye	0.000739	0.0272	0	1	56	0.00253	0.0502	0	1	466
C70–72 Central nervous system	0.0438	0.205	0	1	3320	0.0464	0.210	0	1	8552
C73 Thyroid gland	0.00263	0.0512	0	1	199	0.0106	0.102	0	1	1956
C81 Hodgkin lymphoma	0.00104	0.0323	0	1	79	0.00426	0.0652	0	1	786
C82–86, C96 Non-Hodgkin lymphoma	0.0261	0.159	0	1	1977	0.0305	0.172	0	1	5616
C88 Immunoproliferative disease	0.000462	0.0215	0	1	35	0.00166	0.0407	0	1	306
C90 Multiple myeloma	0.0206	0.142	0	1	1557	0.0124	0.111	0	1	2292
C91–95 Leukaemia	0.0273	0.163	0	1	2068	0.0269	0.162	0	1	4968
$\textit{No. of observations}$	75,741					184,344

**Table 8:** Effect of testing for biomarkers on premature mortality and survival, with alternative minimum number of packages sold

	logPYLL75			logPYLL65			3-year survival		
	50	100	500	50	100	500	50	100	500
	Packages	Packages	Packages	Packages	Packages	Packages	Packages	Packages	Packages
Biomarker(s) available	− 0.211*** (0.0712)	− 0.202*** (0.0701)	− 0.192*** (0.0645)	− 0.267*** (0.0919)	− 0.254*** (0.0918)	− 0.253*** (0.0837)	0.061** (0.0228)	0.057** (0.0227)	0.054** (0.0237)
Biomarker(s) $\times$ drugs	0.004 (0.0033)	0.004 (0.0033)	0.004 (0.0031)	0.005 (0.0039)	0.005 (0.0040)	0.006 (0.0040)	− 0.004*** (0.0008)	− 0.004*** (0.0008)	− 0.004*** (0.0010)
# Drugs available	− 0.021** (0.0092)	− 0.019** (0.0089)	− 0.030** (0.0125)	− 0.025* (0.0130)	− 0.021 (0.0130)	− 0.037** (0.0163)	0.008*** (0.0023)	0.008*** (0.0027)	0.008 (0.0047)
Biomarker marginal effect	− 0.147*** (0.0403)	− 0.146*** (0.0405)	− 0.141*** (0.0379)	− 0.189*** (0.0552)	− 0.190*** (0.0557)	− 0.182*** (0.0511)	0.009 (0.0146)	0.008 (0.0148)	0.009 (0.0158)
*R*-square	0.775	0.774	0.775	0.771	0.770	0.772	0.205	0.205	0.205
No. of obs	71656	71656	71656	36040	36040	36040	184344	184344	184344

Ordinary least squares (OLS) coefficients with cancer type fixed effects and year of diagnosis fixed effects. Standard errors in parentheses are heteroskedastic robust and corrected for clustering at the cancer type level. Controls include age at diagnosis, gender, stage, county of residence, and prevalence. Biomarker marginal effect reports the average marginal effect, where the effects are calculated for each observation and then averaged.

*$$p<0.1$$, **$$p<0.05$$, ***$$p<0.01$$

**Table 9:** Effect of testing for biomarkers on survival (logistic regression)

	(1)
	3-year survival
Biomarker(s) available	0.375*** (0.1258)
Biomarker(s) $\times$ drugs	− 0.017*** (0.0056)
# Drugs available	0.031** (0.0154)
Biomarker marginal effect	0.014** (0.0070)
No. of obs	184,344

Logistic regression coefficients with cancer type fixed effects and year of diagnosis fixed effects. Standard errors in parentheses are heteroskedastic robust and corrected for clustering at the cancer type level. Controls include age at diagnosis, gender, stage, county of residence, and prevalence. Biomarker marginal effect reports the average marginal effect, where the effects are calculated for each observation and then averaged.

*$$p<0.1$$, **$$p<0.05$$, ***$$p<0.01$$

**Table 10:** Effect of testing for biomarkers on premature mortality and survival, with sample restricted to cancer types with biomarker

	logPYLL75	logPYLL65	3-year survival
Biomarker(s) available	− 0.287**	− 0.374**	0.026
	(0.0599)	(0.0897)	(0.0197)
	[0.0120]	[0.0150]	[0.3544]
Biomarker(s) $\times$ drugs	0.009**	0.012*	− 0.002*
	(0.0021)	(0.0033)	(0.0006)
	[0.0460]	[0.0661]	[0.0631]
# Drugs available	− 0.037**	− 0.051**	0.002
	(0.0073)	(0.0113)	(0.0052)
	[0.0370]	[0.0320]	[0.8348]
Biomarker marginal effect	− 0.137***	− 0.167***	− 0.013
	(0.0316)	(0.0437)	(0.0111)
*R*-square	0.790	0.787	0.265
No. of obs	35,428	17,985	82,567

Ordinary least squares (OLS) coefficients with cancer type fixed effects and year of diagnosis fixed effects. Standard errors in parentheses are heteroskedastic robust and corrected for clustering at the cancer diagnosis level (9 clusters). In square brackets, we report the wild cluster bootstrap *p* values generated using the boottest command in Stata 15 [28], with clustering at the cancer type level. Controls include age at diagnosis, gender, stage, county of residence, and prevalence. Biomarker marginal effect reports the average marginal effect, where the effects are calculated for each observation and then averaged.

*$$p<0.1$$, **$$p<0.05$$, ***$$p<0.01$$

**Table 11:** Effect of biomarker-guided and nonguided therapies on premature mortality and survival excluding control variables

	logPYLL75		logPYLL65		3-year survival	
	(1)	(2)	(3)	(4)	(5)	(6)
Biomarker(s) available	0.043 (0.0530)	− 0.191*** (0.0597)	0.064 (0.0481)	− 0.213*** (0.0643)	0.078*** (0.0248)	0.076*** (0.0227)
Biomarker(s)$\times$drugs	− 0.003 (0.0022)	0.004 (0.0037)	− 0.004 (0.0024)	0.003 (0.0037)	− 0.004*** (0.0009)	− 0.004*** (0.0008)
# Drugs available	− 0.004 (0.0062)	− 0.044*** (0.0094)	− 0.011 (0.0093)	− 0.052*** (0.0139)	0.007*** (0.0021)	0.007*** (0.0020)
Age at diagnosis	No	Yes	No	Yes	No	Yes
*R*-square	0.006	0.762	0.008	0.751	0.015	0.035
No. of obs	71,656	71,656	36,040	36,040	184,344	184,344

Ordinary least squares (OLS) coefficients with cancer type fixed effects and year of diagnosis fixed effects. Standard errors in parentheses are heteroskedastic robust and corrected for clustering at the cancer type level.

*$$p<0.1$$, **$$p<0.05$$, ***$$p<0.01$$

**Table 12:** Effect of testing for biomarkers premature mortality and survival, excluding the Biomarker main effect

	logPYLL75	logPYLL65	3-year survival
Biomarker(s) $\times$ drugs	− 0.004** (0.0016)	− 0.005** (0.0022)	− 0.002*** (0.0004)
# Drugs available	− 0.017** (0.0081)	− 0.020 (0.0121)	0.007** (0.0027)
Biomarker marginal effect	− 0.053** (0.0219)	− 0.067** (0.0313)	− 0.024*** (0.0059)
*R*-square	0.774	0.769	0.205
No. of obs	71,656	36,040	184,344

Ordinary least squares (OLS) coefficients with cancer type fixed effects and year of diagnosis fixed effects. Standard errors in parentheses are heteroskedastic robust and corrected for clustering at the cancer type level. Controls include age at diagnosis, gender, stage, county of residence, and prevalence. Biomarker marginal effect reports the average marginal effect, where the effects are calculated for each observation and then averaged.

*$$p<0.1$$, **$$p<0.05$$, ***$$p<0.01$$

**Table 13:** Effect of biomarker testing on premature mortality and survival with different age thresholds

	30–82 years		30–50 years		50–82 years	
	logPYLL82	3-year surv	logPYLL50	3-year surv	logPYLL82	3-year surv
Biomarker(s) available	− 0.188*** (0.0645)	0.061** (0.0233)	− 0.406*** (0.1355)	0.029 (0.0184)	− 0.222*** (0.0814)	0.060** (0.0229)
Biomarker(s) $\times$ drugs	0.004 (0.0031)	− 0.004*** (0.0009)	0.009* (0.0052)	− 0.002*** (0.0006)	0.005 (0.0037)	− 0.004*** (0.0009)
# Drugs available	− 0.016** (0.0070)	0.008*** (0.0027)	− 0.020 (0.0205)	0.003 (0.0018)	− 0.019** (0.0082)	0.008** (0.0032)
Biomarker marginal effect	− 0.135*** (0.0378)	0.010 (0.0154)	− 0.273*** (0.0900)	0.005 (0.0111)	− 0.157*** (0.0480)	0.009 (0.0160)
*R*-square	0.779	0.220	0.776	0.161	0.809	0.220
No. of obs	103,241	223,889	6434	35,335	92,902	191,987

Ordinary least squares (OLS) coefficients with cancer type fixed effects and year of diagnosis fixed effects. Standard errors in parentheses are heteroskedastic robust and corrected for clustering at the cancer type level. Controls include age at diagnosis, gender, stage, county of residence, and prevalence. Biomarker marginal effect reports the average marginal effect, where the effects are calculated for each observation and then averaged.

*$$p<0.1$$, **$$p<0.05$$, ***$$p<0.01$$

**Table 14:** Effect of testing for biomarkers on premature mortality and survival, distant and localized cancer spreads

	logPYLL75		logPYLL65		3-year survival	
	(1)	(2)	(3)	(4)	(5)	(6)
	Distant	Localized	Distant	Localized	Distant	Localized
Biomarker(s) available	− 0.210*** (0.0477)	− 0.211*** (0.0553)	− 0.237*** (0.0562)	− 0.280*** (0.0913)	0.005 (0.0325)	0.036 (0.0246)
Biomarker(s) $\times$ drugs	0.009*** (0.0018)	0.007** (0.0030)	0.008*** (0.0021)	0.009*** (0.0032)	0.001 (0.0016)	− 0.002** (0.0009)
# Drugs available	− 0.021*** (0.0061)	− 0.034*** (0.0080)	− 0.028*** (0.0065)	− 0.044*** (0.0082)	− 0.008* (0.0046)	0.005 (0.0036)
Biomarker marginal effect	− 0.098*** (0.0283)	− 0.138*** (0.0445)	− 0.131*** (0.0322)	− 0.185*** (0.0685)	0.014 (0.0188)	0.009 (0.0154)
*R*-square	0.790	0.757	0.787	0.770	0.026	0.031
No. of obs	31,582	9466	16,303	4809	33,575	72,998

Ordinary least squares (OLS) coefficients with cancer type fixed effects and year of diagnosis fixed effects. Standard errors in parentheses are heteroskedastic robust and corrected for clustering at the cancer type level. Controls include age at diagnosis, gender, stage, county of residence, and prevalence. Biomarker marginal effect reports the average marginal effect, where the effects are calculated for each observation and then averaged.

*$$p<0.1$$, **$$p<0.05$$, ***$$p<0.01$$

## Abbreviations

BRAF: B-Raf proto-oncogene serine/threonine kinase
CCBIO: Centre for cancer biomarkers
EGFR: Epidermal growth factor recep2tor
EMA: European Medicines Agency
EUR: Euro
FDA: Food and Drug Administration
HER2: Human epidermal growth factor receptor 2
KRAS: Kirsten rat sarcoma
NOK: Norwegian Krone
NRAS: Neuroblastoma RAS viral oncogene
QALY: Quality-adjusted life-year
WHO-ICD: World Health Organization, International Classification of Diseases

## Footnotes

1 Lead-time bias occurs if improvements in screening tests for some cancer types lead to earlier diagnosis [18]). This could increase the survival time because the patient is diagnosed earlier and not necessarily because the patient receives more and/or better treatment.

2 In the robustness checks section of the article, we test whether other package cut-offs affects our results.

3 This ideal measure should contain all cancer drugs taken, including those consumed at the hospital, since many cancer drugs must be applied intravenously by a health practitioner. These data are unfeasible to obtain from any Norwegian central registry.

4 The data from the Cancer Registry of Norway contain the results of some biomarker tests (estrogen and progesterone receptors, and HER2 for breast cancer, EGFR and KRAS for colorectal cancer, and ALK for lung cancer) for some patients in a simple form (for example, whether the patient is HER2-positive or HER2-negative). The completeness of these data is, however, quite low, due to the clinician’s low degree of reporting. Therefore, this information is not used in the analysis.

5 The limit of 75 years has been chosen for the calculations in OECD Health Data. Additionally, the age thresholds of 75 and 65 have been widely used in the literature [16, 18, 19].

6 The summary statistics of the cancer types are available in the appendix, Table 7.

7 For the breast cancer cases, the test for estrogen and progesterone receptors was already available before our period of study, while the HER2 test was introduced in 2005. Therefore, for breast cancers, the *Biomarker* variable is equal to one when the HER2 test is available.

8 Prevalence was provided by the Cancer Registry of Norway.

9 We also run model (1) on a categorical variable of biomarker tests, as it can only take values of 0, 1, or 2 tests available per cancer type. The results are available upon request.

10 Taking derivatives of model (1) with respect to *Biomarker*, we find that the marginal effect of biomarker testing is $\frac{{\partial Y}}{{\partial {\text {Biomarker}}}} = \beta _{1} + \beta _{3} {\text {Drugs}}$. The tables report the average marginal effects, which is found by calculating the marginal effects at each value observed in the dataset and then calculating the average.

11 Setting these criteria as a limit on the total sales in terms of the pharmacy purchase price is unreasonable since the price per unit varies greatly across drugs. The amount of active ingredient is presented differently depending on the type of drug, where the sales are reported either in grams, milligrams, or DDDs (Defined Daily Doses). Moreover, the sales of some anti-cancer vaccines are not presented in the amount of active ingredient. Therefore, total sales in the number of packages was the criterion chosen.

12 The case of two new drugs Opdivo and Keytruda is an example. The drugs are very similar but only work for a minority of patients—those whose tumors have high levels of a biomarker known as PD-L1. Only Keytruda required the patients to have prior testing, while Opdivo did not. However, Opdivo’s sales increased much more rapidly than its competitor’s since oncologists were not required to order the biomarker test first [6].

13 The biomarker positive patients are a small population for ALK+ and EGFR+, but not for BRAF+ and RAS+.

14 Our data do not allow us to observe whether the prognosis of each patient has changed over time. Information on the stage of disease in our dataset is only available at the time of diagnosis.

## References

[1] Andrade LF, Sermet C, Pichetti S (2014). Entry time effects and follow-on drug competition. Eur. J. Health Econ..

[2] Behl AS, Goddard KA, Flottemesch TJ, Veenstra D, Meenan RT, Lin JS, Maciosek MV (2012). Cost-effectiveness analysis of screening for KRAS and BRAF mutations in metastatic colorectal cancer. JNCI J. Natl. Cancer Inst..

[3] Bleyer A, Barr R, Hayes-Lattin B, Thomas D, Ellis C, Anderson B (2008). The distinctive biology of cancer in adolescents and young adults. Nat. Rev. Cancer.

[4] Domingues B, Lopes JM, Soares P, Pópulo H (2018). Melanoma treatment in review. ImmunoTargets Ther..

[5] Dubois, P., Kyle, M.: The Effects of Pharmaceutical Innovation on Cancer Mortality Rates. TSE Working Paper No. 16-688 (2016)

[6] Financial Times.: “Merck plays long game in precision medicine battle”. https://www.ft.com/content/1ee2402a-6dfd-11e6-a0c9-1365ce54b926 (2016). Accessed 30 Aug

[7] Fisher ES, Welch HG (1999). Avoiding the unintended consequences of growth in medical care: how might more be worse?. Jama.

[8] Fiva JH, Hægeland T, Rønning M, Syse A (2014). Access to treatment and educational inequalities in cancer survival. J. Health Econ..

[9] Garrison LP, Austin MJF (2006). Linking pharmacogenetics-based diagnostics and drugs for personalized medicine. Health Aff..

[10] Garrison LP, Towse A, Culyer AJ (2014). Personalized medicine: pricing and reimbursement policies as a potential barrier to development and adoption, economics of. Encyclopedia of Health Economics.

[11] Greene, W., Han, C., Schmidt, P.: The bias of the fixed effects estimator in nonlinear models. Unpublished Manuscript, Stern School of Business, NYU, 29 (2002)

[12] Lange A, Prenzler A, Frank M, Golpon H, Welte T, von der Schulenburg JM (2014). A systematic review of the cost-effectiveness of targeted therapies for metastatic non-small cell lung cancer (NSCLC). BMC Pulm. Med..

[13] Li M, Basu A, Bennette CS, Veenstra DL, Garrison LP (2019). Do cancer treatments have option value? Real-world evidence from metastatic melanoma. Health Econ..

[14] Lichtenberg FR (2009). The effect of new cancer drug approvals on the life expectancy of American cancer patients, 1978–2004. Econ. Innov. New Technol..

[15] Lichtenberg FR (2012). Contribution of pharmaceutical innovation to longevity growth in Germany and France, 2001–2007. Pharmacoeconomics.

[16] Lichtenberg FR (2013). The impact of new (orphan) drug approvals on premature mortality from rare diseases in the United States and France, 1999–2007. Eur. J. Health Econ..

[17] Lichtenberg FR (2015). The impact of pharmaceutical innovation on premature cancer mortality in Canada, 2000–2011. Int. J. Health Econ. Manag..

[18] Lichtenberg FR (2015). The impact of pharmaceutical innovation on premature cancer mortality in Switzerland, 1995–2012. Eur. J. Health Econ..

[19] Lichtenberg FR (2017). The impact of pharmaceutical innovation on cancer mortality in Belgium, 2004–2012. Forum Health Econ. Policy.

[20] Lichtenberg FR (2017). The impact of pharmaceutical innovation on cancer mortality in Mexico, 2003–2013. Latin Am. Econ. Rev..

[21] Lichtenberg FR (2020). How cost-effective are new cancer drugs in the US?. Expert Rev Pharmacoeconomics Outcomes Res.

[22] Helsedirektoratet.: Nasjonal strategi for persontilpasset medisin i helsetjenesten. https://helsedirektoratet.no/publikasjoner/strategi-for-persontilpasset-medisin-i-helsetjenesten (2016)

[23] Naylor S, Cole T (2010). Overview of companion diagnostics in the pharmaceutical industry. Drug Discov. World.

[24] Oosterhoff M, van der Maas ME, Steuten LM (2016). A systematic review of health economic evaluations of diagnostic biomarkers. Appl. Health Econ. Health Policy.

[25] Oppong R, Jit M, Smith RD, Butler CC, Melbye H, Mölstad S, Coast J (2013). Cost-effectiveness of point-of-care C-reactive protein testing to inform antibiotic prescribing decisions. Br. J. Gen. Pract..

[26] Romanus D, Cardarella S, Cutler D, Landrum MB, Lindeman NI, Gazelle GS (2015). Cost-effectiveness of multiplexed predictive biomarker screening in non-small-cell lung cancer. J. Thorac. Oncol..

[27] Romer PM (1990). Endogenous technological change. J. Polit. Econ..

[28] Roodman, D., MacKinnon, J.G., Nielsen, M.O., Webb, M.D.: Fast and wild: bootstrap inference in Stata using boottest (No. 1406) (2018)

[29] Russo A, Ficili B, Candido S, Pezzino FM, Guarneri C, Biondi A, Travali S, McCubrey JA, Spandidos DA, Libra M (2014). Emerging targeted therapies for melanoma treatment (review). Int. J. Oncol..

[30] Seo MK, Cairns J (2018). Do cancer biomarkers make targeted therapies cost-effective? A systematic review in metastatic colorectal cancer. PLoS One.

[31] Temple RJ, Himmel MH (2002). Safety of newly approved drugs: implications for prescribing. JAMA.

[32] The Economist.: Treating cancer: progress on many fronts. https://www.economist.com/technology-quarterly/2017-09-16/treating-cancer (2017). Accessed 16 Sept

